# The signalling lipid PI3,5P_2_ is essential for timely mitotic exit

**DOI:** 10.1098/rsob.230125

**Published:** 2023-09-27

**Authors:** Mariam Huda, Seyma Nur Bektas, Baris Bekdas, Ayse Koca Caydasi

**Affiliations:** Department of Molecular Biology and Genetics, Koç University, Istanbul, Turkey

**Keywords:** PI3, signalling lipid, phosphoinositide, mitosis, mitotic exit network, mitotic exit

## Abstract

Coordination of mitotic exit with chromosome segregation is key for successful mitosis. Mitotic exit in budding yeast is executed by the mitotic exit network (MEN), which is negatively regulated by the spindle position checkpoint (SPOC). SPOC kinase Kin4 is crucial for SPOC activation in response to spindle positioning defects. Here, we report that the lysosomal signalling lipid phosphatidylinositol-3,5-bisphosphate (PI3,5P_2_) has an unanticipated role in the timely execution of mitotic exit. We show that the lack of PI3,5P_2_ causes a delay in mitotic exit, whereas elevated levels of PI3,5P_2_ accelerates mitotic exit in mitotic exit defective cells. Our data indicate that PI3,5P_2_ promotes mitotic exit in part through impairment of Kin4. This process is largely dependent on the known PI3,5P_2_ effector protein Atg18. Our work thus uncovers a novel link between PI3,5P_2_ and mitotic exit.

## Graphical abstract

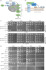

## 1. Introduction

Mitotic exit is a critical step in the cell cycle in which a cell exits mitosis and enters the subsequent G1 phase. In budding yeast, mitotic exit is triggered by the mitotic exit network (MEN), which is a signalling pathway that shares close relationship with the Hippo pathway in animal cells [1–3]. Precise regulation of the MEN is critical to ensure that cells accurately exit mitosis, maintain their genome integrity, and prevent aneuploidy [4–7].

**Figure 1.**
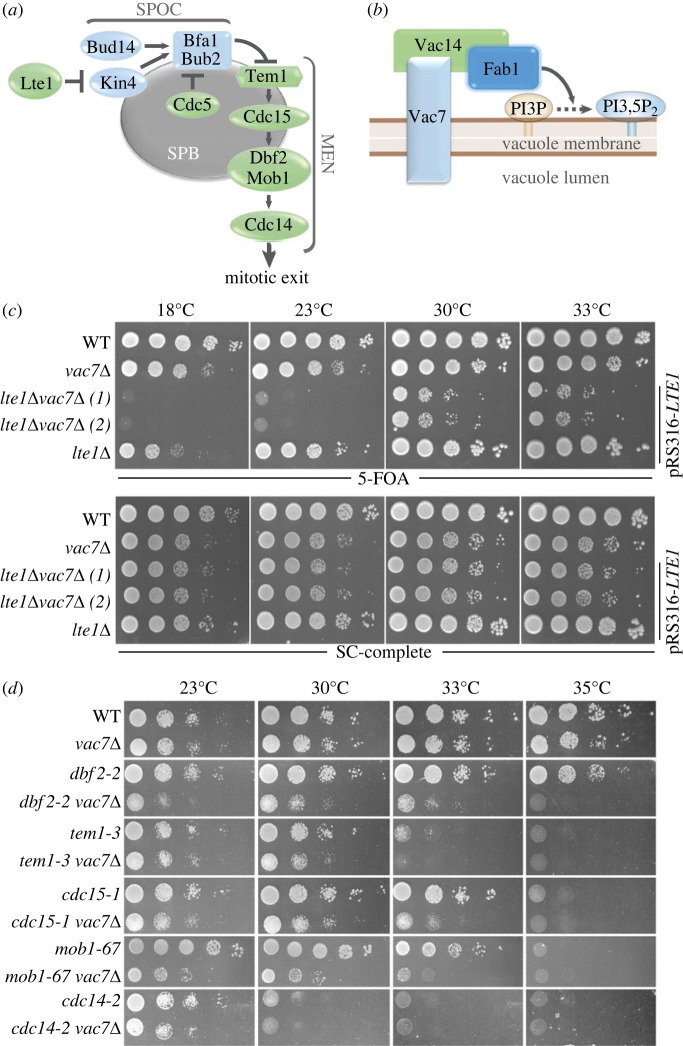
Deletion of *VAC7* causes growth impairment in mitotic exit defective cells. (*a*,*b*) Schematic diagram showing MEN and SPOC pathways (*a*) and PI3,5P2 synthesis (*b*) including the localization of the components. Blunt arrows pointed arrows and the dashed arrow indicate inactivation, activation and biochemical reaction direction, respectively. (*c,d*) Genetic interactions between *vac7*Δ and *lte1*Δ (ESM356, FAY145, SEY023, SEY025, SEY075) (*c*), *vac7*Δ and *men-ts* mutants (MHY013, MHY062, MHY036, MHY063, MHY014, MHY071, MHY015, MHY072, MHY011, MHY076, MHY012, MHY061) (*d*) are analysed by plasmid shuffling. Serial dilutions of indicated strains were spotted on indicated plates and grown at corresponding temperatures. 5-Fluoroorotic acid (5-FOA) plates negatively select for the *URA3*-based plasmids (pRS316 containing the *LTE1* in *c*, pRS416 containing the *VAC7* in *d*). Thus, only cells that have lost these plasmids can grow on 5-FOA plates where genetic interactions can be observed.

The MEN is driven by a Ras-like GTPase Tem1, which is located near the top of the pathway [2,8–10] (figure 1*a*). Activation of Tem1 triggers a kinase cascade comprising Cdc15 and Dbf2-Mob1 kinases [11–14]. Dbf2-Mob1, in turn, promotes the full release of the phosphatase Cdc14 from the nucleolus [15,16] (figure 1*a*). The conserved phosphatase Cdc14 drives cells out of mitosis through dephosphorylation of key CDK targets and eventually inactivation of the mitotic CDK [17].

Faithful mitosis requires mitotic exit occur only after correct segregation of chromosomes to the daughter cell. The spindle position checkpoint (SPOC) ensures this by inhibiting the MEN when the nucleus fails to reach to the daughter cell. At the very downstream, SPOC acts through the Bfa1-Bub2 GAP complex, which inactivates Tem1 by keeping Tem1 in the GDP bound form [18] (figure 1*a*). Both MEN and SPOC use spindle pole bodies (SPB, yeast equivalent of centrosomes) as a scaffold [19–22] (figure 1*a*). When the mitotic spindle aligns correctly, Bfa1-Bub2 and Tem1 localize to the SPB, which migrates to the daughter cell (dSPB) [23,24]. At the dSPB, Cdc5, a polo-like kinase, inactivates Bfa1-Bub2 by phosphorylating Bfa1 [25] (figure 1*a*). in the case of spindle mispositioning, Kin4 kinase localizes to both SPBs and phosphorylates Bfa1 to activate the Bfa1-Bub2 GAP complex [26–28] (figure 1*a*). Kin4 phosphorylation causes dissociation of the GAP complex from SPBs and prevents Cdc5 phosphorylation of Bfa1 [27,29–32]. In parallel, Protein Phosphatase 1 (Glc7), with its regulatory unit Bud14, counteracts Cdc5 phosphorylation of Bfa1 [33] (figure 1*a*). Taken together, mispositioning of the mitotic spindle activates Bfa1-Bub2 and assures MEN inhibition until the spindle position is corrected.

In addition to the MEN inhibitory SPOC mechanism, MEN promoting mechanisms assure timely mitotic exit. One of those mechanisms is dependent on the cdc Fourteen Early Anaphase Release (FEAR) network which is activated at the time of anaphase onset [34,35]. FEAR network primes the MEN through de-phosphorylation of Cdc15, Mob1-Dbf2 and Bfa1, acting as a timer to support MEN activation after anaphase onset [36–38]. Another MEN promoting mechanism is located in the bud. The bud cortex localized Guanine Nucleotide Exchange Factor (GEF) Lte1 creates a mitotic exit activating zone in the daughter cell compartment at least in part by inhibiting Kin4 in the bud [39–43]. Concomitant absence of Lte1 and FEAR results in a late anaphase arrest, which can be relieved by deletion of SPOC components [26,43].

In multiple independent genome-wide high-throughput genetic screens, we and others identified *VAC7*, *VAC14* and *FAB1* among genes that became essential for colony growth in the absence of known mitotic exit regulators [36,44–46]. Vac7 and Vac14 are activators of the conserved lipid kinase Fab1 (PIKfyve in human), which catalyzes the synthesis of phosphatidylinositol-3,5-bisphosphate (PI3,5P_2_) through phosphorylation of phosphatidylinositol-3-phosphate (PI3P) [47–54] (figure 1*b*). Vac7, Vac14 and Fab1 are primarily localized on the membrane of the vacuole/lysosome [50,55,56] and Fab1 also localizes to some extend on the membranes of the signalling endosomes [57] (figure 1*b*). PI3,5P_2_ is found at the lysosomal and endosomal membranes, and function in vacuole/lysosome structure/function, stress response, autophagy, transcriptional regulation and membrane trafficking [58–61]. Through its function in regulation of Target of Rapamycin Complex 1 (TORC1), PI3,5P_2_ was also implemented in cell cycle commitment [62,63]. PI3,5P_2_ is one of the least abundant and least understood phosphoinositides [64–66]. Defects in PI3,5P_2_ production are associated with neurological diseases such as amyotrophic lateral sclerosis [67–71] and cancer [72–74].

Here, we reveal that the rare phosphoinositide PI3,5P_2_ has a mitotic exit promoting function in budding yeast. Disruption of the Fab1-Vac7-Vac14 complex led to delayed mitotic exit and lethality in cells with reduced mitotic exit activity. Conversely, overproduction of PI3,5P_2_ via a hyperactive *FAB1* allele (*fab1-ha*) rescued the prolonged anaphase and lethality of mitotic exit mutants. Moreover, we found that PI3,5P_2_ overproduction resulted in the mis-localization of the mitotic exit inhibitor Kin4 at the vacuole periphery, impairment of Kin4 function, and SPOC failure. Lack of PI3,5P_2_, on the other hand, disrupted asymmetric distribution of Kin4 among the mother and daughter cytoplasm, resulting in more Kin4 in the daughter cell compartment. Our data suggest that modulation of Kin4 by PI3,5P_2_ required the PI3,5P_2_ binding protein Atg18. We further provide evidence indicating that PI3,5P_2_ employs both Atg18-Kin4 dependent and Atg18-Kin4 independent mechanisms in promoting mitotic exit. We thus propose a model in which the signalling lipid PI3,5P_2_ contributes to a timely mitotic exit at least in part via the regulation of Kin4 function.

## 2. Results

### 2.1. Impairment of Fab1-Vac7-Vac14 complex causes lethality in cells with mitotic exit defects

PI3,5P_2_ production depends on the PI3,5P_2_ regulatory complex composed of the lipid kinase Fab1 and its regulators Vac7, Vac14 [47,48,50,51,53,54,75] (figure 1*b*). Deletion of either component results in diminishment of PI3,5P_2_ synthesis [49,50]. We asked whether disruption of the Fab1-Vac7-Vac14 complex, and hence lack of PI3,5P_2_, impairs growth of mitotic exit defective cells. For this, first, we deleted *VAC7* in *lte1*Δ cells. Lte1 is a mitotic exit activator (figure 1*a*) that becomes essential for mitotic exit at lower temperatures (less than 16 C) [43]. Deletion of *VAC7* in *lte1*Δ cells caused lethality at 18°C and 23°C, and growth impairment at 30°C and above temperatures (figure 1*c*; electronic supplementary material, figure S1A; table 1). Deletion of *VAC14* in *lte1*Δ cells also caused similar growth lethality (electronic supplementary material, figure S1B; table 1). As deletion of *FAB1* is lethal [76] we exploited a temperature sensitive allele of *FAB1* (*fab1–2*) [54] to analyse the effect of Fab1 kinase on the growth of *lte1*Δ cells. Whereas *fab1–2* is lethal at 37°C, deletion of *LTE1* in *fab1–2* cells caused lethality at 35°C and slow growth at 33°C (electronic supplementary material, figure S1C; table 1). These temperatures corresponded to the semi-permissive temperatures of *fab1–2* as judged from the vacuole enlargement phenotype and growth of *fab1–2* cells at different temperatures (electronic supplementary material, figure S1D).

**Table 1.** Summary of genetic interactions. SL: synthetic lethality; SR: synthetic growth rescue; NI: no detectable genetic interaction. Empty cells show untested interactions.

	***vac7***Δ	***vac14***Δ	***fab1–2***	***atg18***Δ	***vps34***Δ	***fab1-ha***	***bfa1***Δ	***kin4***Δ
*lte1*Δ	SL	SL	SL			SR		
*tem1–3*	SL			SL		SR		
*mob1–67*	SL			SL	SL	SR		
*cdc15–1*	SL			SL	SL			
*dbf2–2*	SL			NI				
*cdc14–2*	SL			NI				
*vac7*Δ						SR		
*vac7*Δ *lte1*Δ						SR	SR	SR
*vac7*Δ *mob1–67*						SR		
*vac7*Δ *tem1–3*						SR		
*vac7*Δ *dbf2–2*						SR		
*vac7*Δ *cdc14–2*						NI		
*vac7*Δ *cdc15–1*						SR		SR
*mob1–67 atg18*Δ						SR		
*Gal1-KIN4*						SR		

We next analysed genetic interactions between Vac7 and other mitotic exit mutants. To this end, we used temperature sensitive mutants of MEN (figure 1*a*) proteins (*men-ts*) [1,33]. Deletion of *VAC7* impaired growth of *mob1–67*, *dbf2–2*, *cdc15–1* and *tem1–3* (figure 1*d* and table 1). These data altogether indicate that cells with mitotic exit defects rely on the Fab1-Vac7-Vac14 complex integrity for their growth.

### 2.2. Deletion of *VAC7* causes mitotic exit delay in *lte1*Δ cells

**Figure 2.**
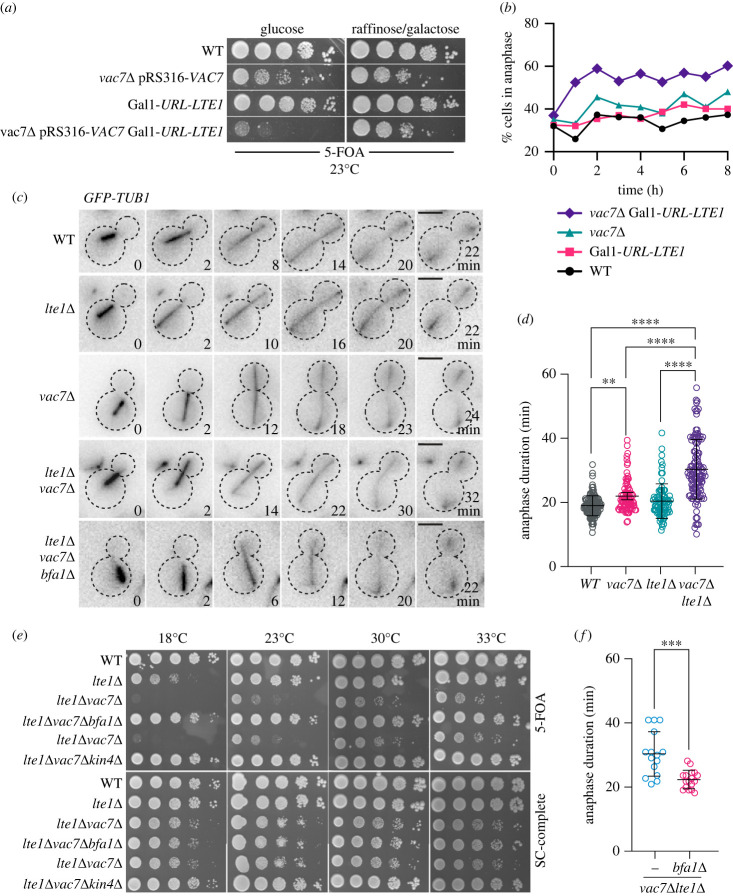
Anaphase duration is longer in *lte1*Δ *vac7*Δ than in other cells. (*a*) Serial dilutions of strains with indicated genotypes (ESM356, SEY254, MHY109, MHY106) were spotted on glucose or galactose containing 5-FOA plates and incubated at 23°C. Note that complementation of *vac7*Δ is lost on these plates. *LTE1-degron* expressed under Gal1 promoter (GAL1-URL-*LTE1*) is depleted on Glucose plates, where the synthetic lethality between *LTE1* and *VAC7* is observed. (*b*) Cells shown in (*a*) were grown to log-phase in galactose containing medium at 23°C and transferred to glucose containing medium (t0), and further cultured. Samples were taken every hour and analysed by microscopy after DAPI staining. At least 100 cells were count at each time point per sample. Percentage of anaphase cells were plotted. A representative graph out of three independent experiment is shown. (*c*) Representative still images from time-lapse series of indicated cells (SEY037, SEY036, BBY024, SEY034, SEY115) at 30°C. *GFP-TUB1* served as a marker for mitotic progression. Time point zero marks 2 min before anaphase onset. Last time points shown are the time of spindle breakdown. Dashed lines show cells' outline. Scale bars: 3 µm. (*d*) Dot plots showing anaphase duration of individual cells (SEY037, SEY036, BBY024, SEY034) at 30°C. Anaphase duration was calculated as the time elapsed between the onset of anaphase and spindle breakdown. Lateral black lines show the mean. Error bars are standard deviation. Sample sizes are 136, 82, 81 and 119 cells for WT, *vac7*Δ, *lte1*Δ and *lte1*Δ*vac7*Δ respectively. Ordinary one-way ANOVA was performed with Tukey's multiple comparison test. ** *p* < 0.01, **** *p* < 0.0001. (*e*) Spot assay that shows deletion of the mitotic exit inhibitors *BFA1* and *KIN4* rescue synthetic lethality of *lte1*Δ *vac7*Δ. Serial dilutions of indicated strains (ESM356, SEY023, SEY025, SEY040, SEY080) were spotted on SC-complete and 5-FOA plates. All *lte1*Δ strains are complemented with pRS316-*LTE1* and thus *lte1*Δ phenotype is observed only on 5FOA plates. (*f*) Dot plots showing anaphase duration of individual cells (SEY034, SEY115) at 30°C. Lateral black lines show the mean. Error bars are standard deviation. Sample sizes are 15 and 17 for *lte1*Δ*vac7*Δ and *lte1*Δ*vac7*Δ respectively. Unpaired two-tailed *t*-test was performed. ***p = 0.0001.

Next, we asked whether *lte1*Δ *vac7*Δ lethality stems from defective mitotic exit. To address this question, we constructed a Gal1-*URL*-*LTE1* strain which contains an N-terminal degron sequence and thus allows for Lte1 depletion in glucose containing media (electronic supplementary material, figure S2A) [77]. In line with lethality of *lte1*Δ *vac7*Δ, Gal1-*URL*-*LTE1 vac7*Δ cells failed to form colonies on glucose containing agar plates at 23°C (figure 2*a*). We reasoned that if the lethality was due to mitotic exit problems, Gal1-*URL*-*LTE1 vac7*Δ population would accumulate cells in anaphase/telophase upon growth in glucose containing medium. To test this, log-phase cultures of Gal1-*URL*-*LTE1 vac7*Δ cells grown in galactose containing medium were transferred into glucose containing medium and percentage of cells with separated nuclei was assessed after the growth medium shift. Unlike wild type, Gal1-*URL*-*LTE1* or *vac7*Δ populations, cells with separated nuclei accumulated in the Gal1-*URL*-*LTE1 vac7*Δ population after 2 h in glucose containing medium (figure 2*b*; electronic supplementary material, figure S2B). This data supports that concomitant loss of Lte1 and Vac7 causes significant defects mitotic exit and/or cytokinesis.

To understand whether mitotic exit is delayed in cells lacking *LTE1* and *VAC7*, we assessed timing of mitotic exit in cells through time lapse microscopy at 30°C. Using GFP-Tub1 as a marker for mitotic spindle we calculated anaphase duration as the time from anaphase onset to mitotic exit, judged by the start of fast spindle elongation and spindle breakdown (figure 2*c,d*). Anaphase took 19–20 min in average in wild type and *lte1*Δ cells, whereas *vac7*Δ cells had slightly longer anaphase (22 min in average) (figure 2*d*). *lte1*Δ *vac7*Δ cells, on the other hand, had dramatically prolonged anaphase (30 min in average) (figure 2*d*), whereas mitotic spindle elongation dynamics were not altered in cells under investigation (electronic supplementary material, figure S3). Further biochemical analysis of cell cycle progression in synchronously dividing cells revealed that *lte1*Δ *vac7*Δ cells had about 10 min delay in mitotic cyclin (Clb2) degradation compared to *lte1*Δ and vac7Δ cells (electronic supplementary material, figure S4). These data altogether are in support of our conclusion that *lte1*Δ *vac7*Δ cells are unable to activate mitotic exit in a timely manner. In line with this notion deletion of mitotic exit inhibitors *BFA1* or *KIN4* rescued lethality (figure 2*e* and table 1) and shortened the prolonged anaphase (figure 2*f*) of *lte1*Δ *vac7*Δ cells.

PI3,5P_2_ has important functions in vacuole physiology [58]. Accordingly, cells lacking Vac7 have enlarged vacuoles (electronic supplementary material, figure S5). We asked whether lethality or prolonged anaphase of *lte1*Δ *vac7*Δ cells stem from possible pleiotropic effects due to vacuole enlargement. If that was the case, *lte1*Δ *vac7*Δ *bfa1*Δ cells that have normal growth and anaphase duration would have normal sized vacuoles. However, we observed that this was not the case. *lte1*Δ *vac7*Δ and *lte1*Δ *vac7*Δ *bfa1*Δ cells had similar vacuole sizes (electronic supplementary material, figure S5), yet only the former had the lethal and prolonged anaphase phenotype (figure 2*e,f*). Thus, enlargement of vacuoles, *per se*, is unlikely to be the cause of lethality and/or mitotic exit defects observed in *lte1*Δ *vac7*Δ cells.

### 2.3. Lack of PI3,5P_2_ causes mitotic exit delay

**Figure 3.**
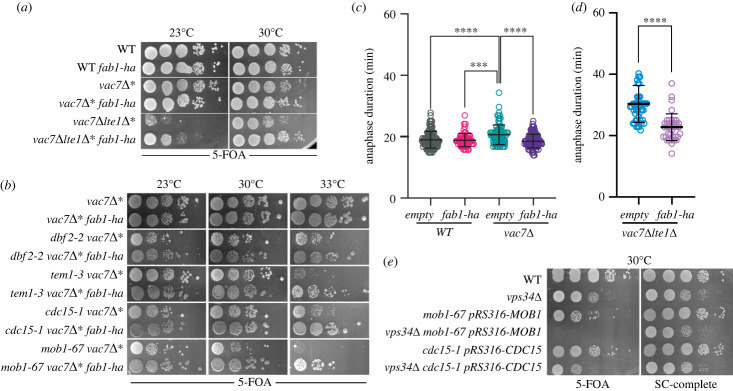
Hyperactive *FAB1* allele (*fab1-ha*) rescues mitotic defects of *lte1*Δ *vac7*Δ and *men-ts vac7*Δ. (*a*,*b*) Spot assays that show *fab1-ha* rescues the synthetic lethality of *lte1*Δ *vac7*Δ (*a*) and *men-ts vac7*Δ (*b*). Indicated strains (in (*a*): SEY228, SEY250, SEY214, SEY213, SEY230 and SEY162; in (*b*): MHY062, MHY079, MHY063, MHY081, MHY071, MHY080, MHY072, MHY082, MHY076 and MHY083) were spotted on 5FOA plates and incubated at corresponding temperatures. *vac7*Δ* and *lte1*Δ* indicates that the strain contains pRS416-*VAC7* and pRS316-*LTE1* respectively. (*c*,*d*) *fab1-ha* rescues anaphase delay of *vac7*Δ (*c*) and *lte1*Δ *vac7*Δ (*d*). Anaphase duration of indicated strains (SEY258, SEY259, SEY264, SEY260, SEY149, SEY143) was calculated and plotted as in figure 2*d*. *empty* denotes for empty plasmid integration control in comparison to the integration of *fab1-ha* bearing integration plasmid into the *leu2* locus. Sample sizes are 107, 66, 94, 121, 49 and 38 cells for *empty*, *fab1-ha*, *vac7*Δ*empty*, *vac7*Δ *fab1-ha*, *lte1*Δ*vac7*Δ*empty* and *lte1*Δ*vac7*Δ*fab1-ha* respectively. Ordinary one-way ANOVA was performed with Tukey's multiple comparison test. *** *p* < 0.001, **** *p* < 0.0001. (*e*) Spot assay that shows deletion of the kinase *VPS34* leads to synthetic lethality with *men-ts*. Indicated strains (MHY013, MHY120–1, ESM1361, YUB002, ESM2282, YUB001) were spotted on 5FOA and SC-complete plates. Note that *mob1–67* and *cdc15–1* are complemented with pRS316-*MOB1* and pRS316-*CDC15* respectively, thus the phenotype is observed on 5FOA plates.

To understand whether the synthetic lethality between mitotic exit activators and Fab1-Vac7-Vac14 complex components is due to lack of PI3,5P_2_, we took advantage of the hyperactive *FAB1* allele (*fab1–14*, *fab1^E1822V F1833L T2250A^*, [49]) here after will be named as *fab1-ha*. *fab1-ha* bypasses the requirement for *VAC7* in PI3,5P_2_ synthesis and thus rescues the PI3,5P_2_ levels and the vacuole enlargement phenotype in *vac7*Δ cells [49] (electronic supplementary material, figure S6). Introduction of *fab1-ha* rescued the lethality of *lte1*Δ *vac7*Δ cells (figure 3*a* and table 1). Growth lethality of *men-ts vac7*Δ was also rescued by the *fab1-ha* allele (figure 3*b* and table 1). In line with these data, *fab1-ha* rescued the mitotic exit delay of *vac7*Δ (figure 3*c*) and *lte1*Δ *vac7*Δ cells (figure 3*d*). These results indicate that Vac7 contributes to a timely mitotic exit through their role in PI3,5P_2_ synthesis.

PI3,5P_2_ is synthesized by phosphorylation of the PI3P inositol head at the fifth position by the PI3P 5-kinase (Fab1). Impairment of Fab1 activity, such as via *VAC7* deletion, indirectly leads to an increase in PI3P levels [50]. We asked whether lethality between the PI3,5P_2_ deficient mutants and mitotic exit defective mutants is a consequence of increased PI3P levels rather than lack of PI3,5P_2_. To address this question, we made use of cells in which *VPS34* is deleted. Vps34 is the PI 3-kinase. Consequently *vps34*Δ cells lack both PI3P and PI3,5P_2_ [64]. We reasoned that if lethality observed in *vac7*Δ *men-ts* cells was due to accumulation of PI3P rather than PI3,5P_2_ deficiency, *vps34*Δ *men-ts* cells that lack both PI3P and PI3,5P_2_ would not be lethal. However, deletion of *VPS34* was lethal in *men-ts* (figure 3*e* and table 1). We thus conclude that it is the lack of PI3,5P_2_ but not increased levels of PI3P that causes lethality in mitotic exit deficient cells.

### 2.4. Elevated PI3,5P_2_ promotes mitotic exit in mitotic exit mutants

**Figure 4.**
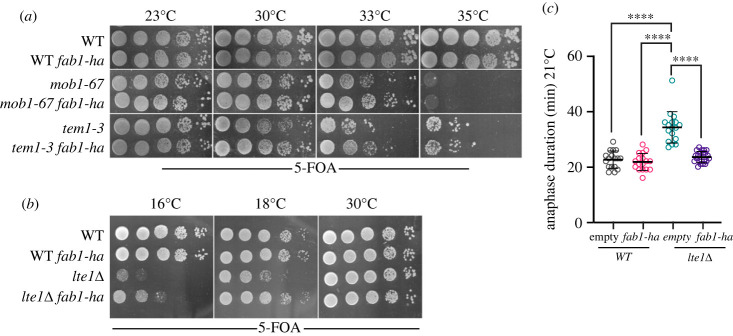
Hyperactive *FAB1* allele (*fab1-ha*) rescues mitotic exit defects of mitotic exit defective mutants. (*a,b*) Spot assays that show *fab1-ha* improves growth of *mob1–67* and *tem1–3* at 33°C (MHY051, MHY050, MHY041, MHY040) (*a*) and *fab1- ha* rescues cold sensitivity of *lte1*Δ (SEY228, SEY250, SEY215, SEY216) (*b*). (*c*) Prolonged anaphase duration of *lte1*Δ at 21°C is rescued by *fab1-ha.* Dot plot shows anaphase duration of individual cells (SEY258, SEY259, SEY262, SEY261). Anaphase duration was calculated and plotted as in figure 2*d*. *empty* denotes for empty plasmid integration control in comparison to the integration of *fab1-ha* bearing integration plasmid into the *leu2* locus. Sample sizes are 17, 22, 17 and 17 cells for *empty*, *fab1-ha*, *lte1*Δ*empty* and *lte1*Δ *fab1-ha* respectively. Ordinary one-way ANOVA was performed with Tukey's multiple comparison test. **** *p* < 0.0001.

*fab1-ha* produces elevated levels of PI3,5P_2_ in the presence of intact Vac7 and Vac14 under basal conditions [49]*.* Accordingly, we asked whether elevating PI3,5P_2_ levels via *fab1-ha* expression could recover the growth of mitotic exit mutants at their semi permissive temperatures. *fab1-ha* improved growth of *mob1–67* and *tem1–3* at 33°C (figure 4*a* and table 1). In addition, *fab1-ha* promoted growth of cold sensitive *lte1*Δ cells. Further analysis of anaphase duration in *lte1*Δ cells at 21°C revealed that *fab1-ha* rescued the anaphase delay of *lte1*Δ cells, whereas it did not accelerate mitotic exit in wild-type cells (figure 4*c*). Thus, overproduction of PI3,5P_2_ promotes mitotic exit in mitotic exit defective cells.

### 2.5. Elevated PI3,5P_2_ alters Kin4 localization

**Figure 5.**
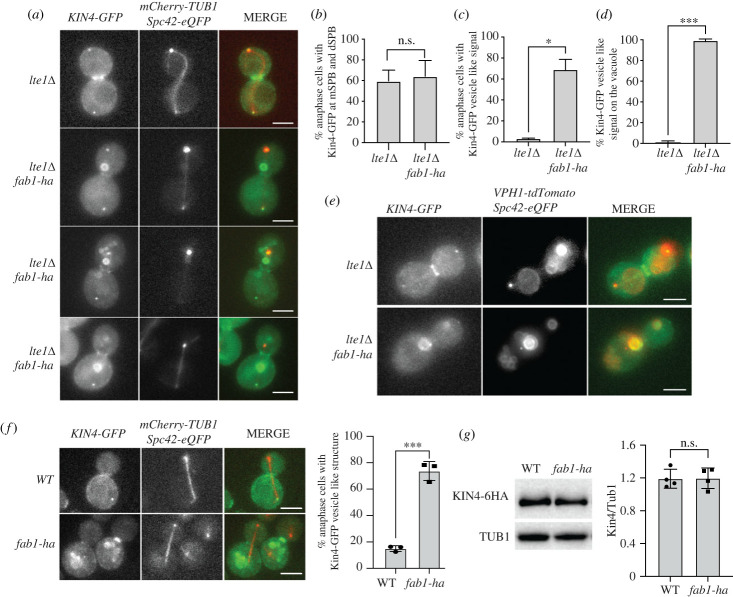
Kin4-GFP localization changes in response to *fab1-ha*. (*a–c*) Kin4-GFP localization in *lte1*Δ (AKY4016), and *lte1*Δ *fab1-ha* (SEY267) cells. Representative still images are shown in (*a*). Percentages of anaphase cells in which Kin4-GFP is present at both SPBs (*b*) and on vesicle like structures (*c*) are plotted. Graphs show the mean of 3 independent experiments. A minimum of 100 cells were counted per sample in each experiment. Error bars are standard error of the mean. Unpaired two-tailed *t*-test was applied. *p = 0.02, n.s: non-significant with *p* = 0.8. Scale bars are 3 µm. (*d,e*) Representative still images and percentage of vesicle-like Kin4-GFP colocalizing with the vacuole marker Vph1-tdTomato in *lte1*Δ (MHY115), and *lte1*Δ *fab1-ha* (MHY116). Graphs show the mean of 3 independent experiments. A minimum of 100 cells were counted per sample in each experiment. Error bars are standard error of the mean. Unpaired two-tailed *t*-test was applied, **p = 0.002. Scale bars are 3 µm. (*f*) Kin4-GFP localization in WT (AKY4005), and *fab1-ha* (AKY4110) cells. Representative still images and percentages of anaphase cells in which Kin4-GFP is present on vesicle like structures. Columns show the mean of 3 independent experiments. Discs and squares show results of individual experiments. Error bars are standard deviation. A minimum of 100 cells were counted per sample in each experiment. Unpaired two-tailed *t*-test was applied, ***p = 0.0002. Scale bars are 3 µm. (*g*) Quantification of relative Kin4 levels in WT (ESM2326), and *fab1-ha* (AKY4115) synchronized in mitosis with nocodazole treatment (on the right) and a representative immunoblot (on the left). Tubulin served as a loading control. Columns show the mean of 4 independent experiments. Discs and squares show results of individual experiments. Error bars are standard deviation. n.s: non-significant with *p* = 0.95 according to unpaired two-tailed t-test.

Lte1 inhibits Kin4 localization in the daughter cell during anaphase. Consequently, Kin4 localizes to the daughter and mother SPBs in *lte1*Δ cells [39,41]. We asked whether *fab1-ha* interferes with Kin4 SPB localization in *lte1*Δ cells. Similar to *lte1*Δ cells, Kin4 localized to both SPBs in *fab1-ha* expressing *lte1*Δ cells during anaphase (figure 5*a,b*). However, unexpectedly, we noticed that Kin4 also localizes to vesicle-like structures in the cytosol of *lte1*Δ cells upon *fab1-ha* expression (figure 5*a,c*).

Considering that PI3,5P_2_ is synthesized on the vacuolar membrane [52,75] (figure 1*b*), we asked whether these vesicle-like structures Kin4 localizes to corresponds to the vacuole. To observe vacuoles, we used Vph1-tdTomato as a vacuolar marker. Kin4-GFP vesicle-like bodies co-localized with vacuole membranes (figure 5*d*,*e*). Similar results were obtained in cells where *LTE1* was not deleted (figure 5*f*, electronic supplementary material, figure S7A). Thus, we conclude that increased PI3,5P_2_ causes Kin4 recruitment to the vacuole membrane.

We reasoned that vacuole membrane localized Kin4 may be degraded by the vacuolar degradation pathway, which may explain how *fab1-ha* promotes mitotic exit. In opposed to this thought, steady state levels of Kin4 were not affected by *fab1-ha* based on western blot analysis (figure 5*g*). Thus, we favour that PI3,5P_2_ promote mitotic exit by a mechanism different than Kin4 protein degradation.

### 2.6. Elevated PI3,5P_2_ hinders Kin4 function

**Figure 6.**
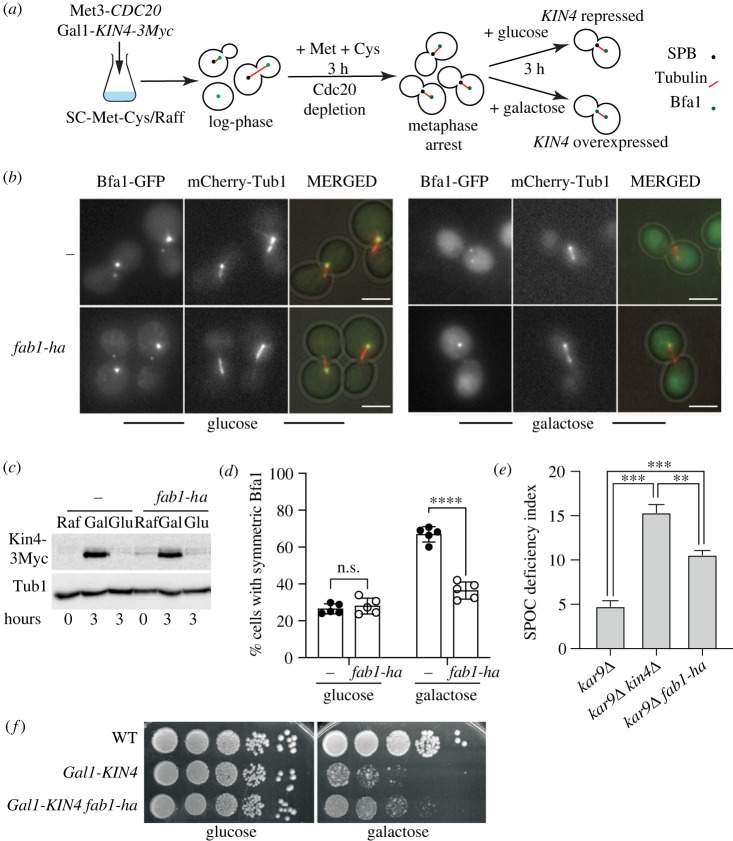
Bfa1 localization in Kin4 overexpressing cells with and without *fab1-ha.* (*a*) Schematic representation of experimental setup. Gal1-*KIN4* Met3-*CDC20* bearing cultures were brought to log-phase in SC-Met-Cys/Raffinose medium, then Methionine and Cystein were added to the cultures to deplete *CDC20*. Metaphase arrest was achieved after 3 h. Finally, either glucose (to represses Kin4 expression) or galactose (to promote Kin4 overexpression) is added and incubated for 3 h until still images were taken. (*b*) Representative still images of metaphase arrested cells without (−) (AKY149) or with *fab1-ha* (MHY148) under Kin4 repressing (glucose) and overexpressing (galactose) conditions. Scale bar: 3 µm. (*c*) Immunoblotting to show *KIN4* expression levels of cells in B. t0 corresponds to metaphase arrest in raffinose (Raf) containing medium after which cells are incubated in galactose (Gal) or glucose (Glu) for 3 hours. (*d*) Percentage of cells with symmetric Bfa1 localization at both SPBs under Kin4 repressing (glucose) and overexpressing (galactose) conditions. Columns show the mean of 5 independent experiments. A minimum of 100 cells were counted per sample in each experiment. Discs and circles show results of individual experiments. Error bars are standard deviation. n.s.: non-significant with *p* = 0.5, ****p < 0.0001 according to unpaired two-tailed *t*-test. (*e*) SPOC deficiency index of *kar9*Δ (AKY346), *kar9*Δ*kin4*Δ (AKY351), and *kar9*Δ *fab1- ha* (MHY149). Columns show the mean of 3 independent experiments. Error bars are standard deviation. A minimum of 100 cells were counted per sample in each experiment. **p < 0.01 and ***p < 0.001 according to ordinary one-way ANOVA. (*f*) Spot assays that show *fab1-ha* (MHY158) improves growth of Gal1*-KIN4* (ESM2244-1). Gal1*-KIN4* overexpression is induced on the Galactose plate. Glucose plate serves as a control for normal growth.

To address functionality of Kin4 upon *fab1-ha* expression, we assessed Bfa1 localization change in response to Kin4 action. Normally, Bfa1 localizes predominantly at one of the SPBs (asymmetric SPB localization) [23,24]. Phosphorylation of Bfa1 by Kin4 causes Bfa1 to localize at both SPBs equally (symmetric SPB localization) [28,30–32]. This Kin4 driven change in Bfa1 localization is essential for inhibition of mitotic exit and can be assayed upon Kin4 overexpression in metaphase arrested cells [78]. As expected, Bfa1 localized asymmetrically to the SPBs in metaphase arrested cells when Kin4 was not overexpressed (figure 6*a,b*, glucose) whereas Bfa1 localized at both SPBs equally (symmetric localization) when Kin4 was overexpressed (figure 6*a,b*, galactose). In the presence of *fab1-ha*, however, Kin4 overexpression did not promote Bfa1 symmetric localization at the SPBs (figure 6*a–d*). Thus, we conclude that when PI3,5P_2_ levels are elevated, Kin4 fails to promote Bfa1 symmetric localization.

Kin4 and Bfa1-Bub2 are essential components of the SPOC that prevents mitotic exit when spindle fails to align correctly. Constitute activation of the SPOC via *KIN4* overexpression causes failure of mitotic exit and thus lethality. We reasoned that if increased levels of PI3,5P_2_ prevents Kin4 activity towards Bfa1, *fab1-ha* would prevent SPOC functionality and rescue lethality of *KIN4* over expression. Supporting this notion, *fab1-ha* expressing cells were SPOC deficient (figure 6*e*). Likewise, *fab1-ha* rescued Kin4 overexpression lethality (figure 6*f*). Altogether, these data suggest that increased levels of PI3,5P_2_ promote mitotic exit of cells through impairment of Kin4 function.

### 2.7. Lack of PI3,5P_2_ disturbs Kin4 cellular distribution

**Figure 7.**
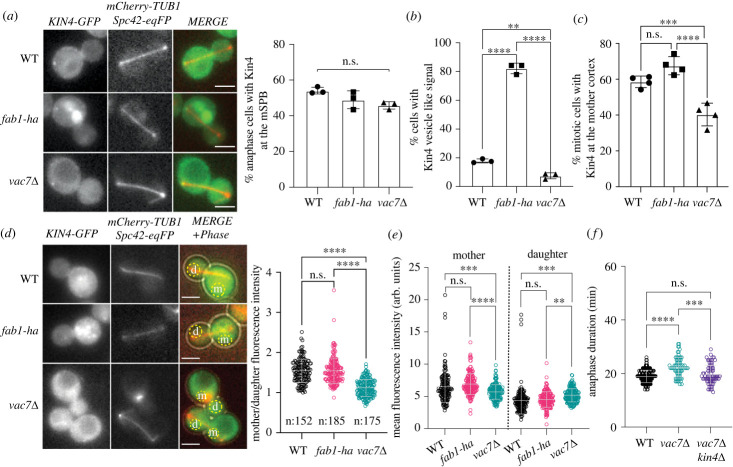
Kin4 cellular distribution is disrupted upon deletion of *VAC7*. (*a–c*) Kin4-GFP localization in WT (AKY4005), *fab1-ha* (AKY4110–1), and *vac7*Δ (AKY4111) cells. Representative still images (*a*, left panel), percentages of anaphase cells with Kin4-GFP at the mother SPB (*a*, right panel), with Kin4-GFP on vesicle like structures (*b*), and on the mother cell cortex (*c*) are plotted. Graphs show the mean of at least 3 independent experiments. A minimum of 100 cells were counted per sample in each experiment. Each data point on the graph represents the result of one experiment. Error bars are standard error of the mean. n.s.: non-significant with *p* > 0.5, **p < 0.01, ***p < 0.001, and ****p < 0.0001 according to ordinary one-way ANOVA. Scale bars are 3 µm. (*d,e*) Kin4-GFP mean fluorescence intensity in mother and daughter cells' cytoplasm of strains described in (*a*). Representative still images that comes from sum projection of 13 z-stacks are shown in (*d*), left panel. The dashed circles in the merged image represent the areas chosen to measure Kin4-GFP mean fluorescence intensity (See materials methods for details). Dot plots of individual mean fluorescence intensities at the mother and daughter cytoplasm (*e*) and the mother-to-daughter ratios of these intensities (*d*, right panel) are plotted. Sample sizes shown in (*d*). Lateral black lines show the mean. Error bars are standard deviation. n.s.: non-significant with *p* > 0.4, **p < 0.01, ***p < 0.001, and ****p < 0.0001 according to ordinary one-way ANOVA. Scale bars are 3 µm. (*f*) Dot plots showing anaphase duration of individual WT (SEY037), *vac7*Δ (BBY024), and *vac7*Δ *kin4*Δ (MHY234) cells at 30°C. Anaphase duration was calculated as the time elapsed between the onset of anaphase and spindle breakdown. Lateral black lines show the mean. Error bars are standard deviation. Sample sizes are 140, 76, and 78 cells for WT, *vac7*Δ, and *vac7*Δ *kin4*Δ respectively. n.s.: non-significant with *p* = 0.5, ***p < 0.001, and ****p < 0.0001 according to ordinary one-way ANOVA.

Analysis of *fab1-ha* bearing cells let us conclude that Kin4 function is impaired in the presence of elevated levels of PI3,5P_2_. In order to understand whether the same mechanism applies under normal levels of PI3,5P_2_, we analysed Kin4 localization in *vac7*Δ cells that fail to produce PI3,5P_2_ [50]. Kin4 localizes to the mother cell cortex throughout the cell cycle and to the SPB that stays in the mother cell compartment (mSPB) transiently during anaphase [26,28]. Kin4 localization at the mSPB of anaphase cells was not significantly changed in *vac7*Δ cells in comparison to the wild-type and *fab1-ha* bearing cells (figure 7*a*). Of importance, vesicle like Kin4 localization that is predominant in *fab1-ha* cells was observed at a much lower frequency in *vac7*Δ cells compared to wild-type cells (figure 7*b*). Kin4 localization at the mother cell cortex was also reduced in *vac7*Δ cells compared to other cell types analysed (figure 7*c*).

Even though Kin4 is mostly localized at the mother cell, it is also present in the daughter cell cytoplasm albeit at lower levels than the mother cell cytoplasm [39]. To assess the distribution of Kin4 at the mother and daughter cell cytoplasm, we measured mean fluorescence intensities of Kin4-GFP at the mother and daughter cell cytoplasm in mitotic cells (spindle length > 1.5 µm) (figure 7*d,e*). In concordance with previous reports, wild-type cells had higher levels of Kin4 at the mother cytoplasm giving rise to a mother to daughter ratio of approximately 1.5 (figure 7*d*). *fab1-ha* bearing cells had a mother to daughter ratio similar to wild-type cells (figure 7*d*). In *vac7*Δ cells, however, mother to daughter ratio of cytoplasmic Kin4-GFP was reduced to approximately 1 (figure 7*c*). Steady state levels of Kin4 were not altered in *vac7*Δ cells (electronic supplementary material, figure S7B), suggesting that the reduction of asymmetric Kin4 distribution among mother and daughter cell cytoplasm does not arise from overall changes in Kin4 levels. Furthermore, based on mean fluorescence intensities, *vac7*Δ cells had less Kin4-GFP in the mother and more Kin4-GFP in the daughter cytoplasm compared to wild type and *fab1-ha* cells (figure 7*e*). These data indicate that PI3,5P_2_ is crucial for Kin4 asymmetric distribution between mother and daughter cell cytoplasm.

We next asked whether increased Kin4 levels at the daughter cell may account for the delay in mitotic exit of *vac7*Δ cells. If this was the case, we would expect *KIN4* deletion to rescue the prolonged anaphase phenotype of *vac7*Δ cells. Supporting this view, deletion of *KIN4* rescued the prolonged anaphase phenotype of *vac7*Δ cells (figure 7*f*). Thus, we conclude that under basal levels, PI3,5P_2_ is important for excluding Kin4 from the daughter cell cytoplasm and thus for timely execution of mitotic exit.

### 2.8. PI3,5P_2_ acts mostly through Atg18 to regulate Kin4 distribution

**Figure 8.**
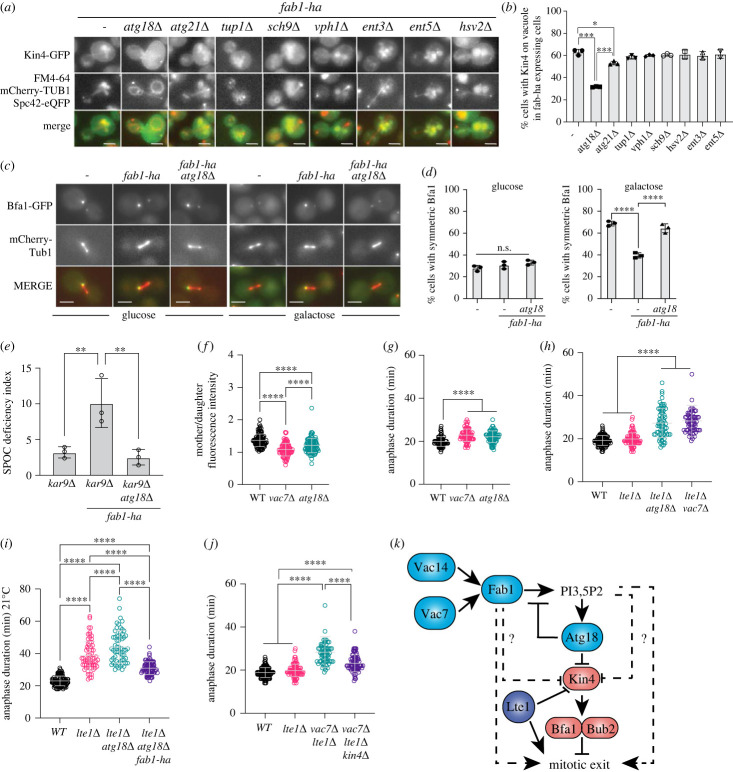
Effect of *fab1-ha* on Kin4 depends on the PI3,5P_2_ effector Atg18. (*a*) Representative still images depicting Kin4 localization in indicated strains with *fab1-ha* (AKY4110, MHY164, MHY165, MHY166, MHY131, MHY129, MHY130, MHY125, and MHY126). FM4–64 dye is used to stain the vacuoles. mCherry-Tub1 and Spc42-eQFP is used as a spindle and SPB markers respectively. Note that vacuole, spindle and SPB markers are observed in red. Scale bar: 3 µm. (*b*) Quantification of Kin4-GFP vacuole localization of strains shown in (*a*). Columns show the mean of independent experiments. Squares, circles and triangles show individual experiments. Error bars are standard deviation. A minimum of 100 cells were counted per sample in each experiment. *p < 0.05, and ***p < 0.001 according to ordinary one-way ANOVA. Only comparisons that yielded significant difference were shown. (*c*,*d*) Representative still images (*c*) and graphs (*d*) showing percentage of cells (AKY116, MHY159, MHY162) with symmetric Bfa1 localization at both SPBs under Kin4 repressing (glucose) and overexpressing (galactose) conditions. Columns show the mean of 3 independent experiments. A minimum of 100 cells were counted per sample in each experiment. Circles, squares and triangles show results of individual experiments. Error bars are standard deviation. n.s.: non-significant, ****p < 0.0001 according to ordinary one-way ANOVA. (*e*) SPOC deficiency indexes of indicated strains (MHY149, AKY346, MHY207). Columns show the mean of 3 independent experiments. A minimum of 100 cells were counted per sample in each experiment. Circles show results of individual experiments. Error bars are standard deviation. **p < 0.01 according to ordinary one-way ANOVA. (*f*) Dot plot showing Kin4-GFP mother-to-daughter fluorescence intensity ratio of the indicated strains (AKY4005, AKY4111, MHY134). A minimum of 100 cells were counted per strain. Lateral black lines show the mean. Error bars are standard deviation. ****p < 0.0001 according to ordinary one-way ANOVA. (*g–j*) Anaphase duration of SEY037, BBY024 and BBY026 grown at 30°C (*g*), SEY037, SEY036, MHY238 and SEY034 grown at 30°C (*h*), SEY037, SEY036, MHY238 and MHY205 grown at 21°C (*i*), SEY037, SEY036, SEY034 and MHY233 grown at 30°C (*j*). A minimum of 88 (*g*), 100 (*h*), 70 (*i*) and 100 (*j*) were counted per strain in corresponding panels. Lateral black lines show the mean. Error bars are standard deviation. ****p < 0.0001 according to ordinary one-way ANOVA. (*k*) Proposed model of mitotic exit control by PI3,5P_2_ signaling. Lines with arrow heads barbed ends indicate positive and negative regulation respectively. Dashed lines show possible scenarios of Atg18-independent mechanisms. See text for details.

To date, there are only few proteins that are known to directly bind PI3,5P_2_. These proteins are Atg18, Atg21, Hsv2, Sch9, Vph1, Tup1, Ent3 and Ent5 [79–83]. We asked whether PI3,5P_2_ dependent modulation of Kin4 localization and function is also dependent on one or more of these proteins that bind PI3,5P_2_. To address this question, we took advantage of Kin4 vacuole localization in *fab1-ha* bearing cells and analysed Kin4 vacuole localization in cells that lack PI3,5P_2_ binding proteins. Vacuole localization of Kin4 was greatly diminished in *atg18*Δ cells, barely reduced in *atg21*Δ cells but remained unchanged in other mutants analysed. (figure 8*a,b*). Furthermore, Kin4's ability to promote symmetric Bfa1 localization at SPBs was rescued upon deletion of *ATG18* in Kin4 overexpressing *fab1-ha* cells (figure 8*c,d*, electronic supplementary material, figure S7C). Similarly, *ATG18* deletion rescued SPOC deficiency of *fab1-ha* cells (figure 8*e*). These experiments altogether suggest that elevated levels of PI3,5P_2_ alters Kin4 localization and inhibits Kin4 function via the PI3,5P_2_ binding protein Atg18.

Next, we analysed contribution of Atg18 to Kin4 asymmetrical distribution when *fab1-ha* is not expressed. Kin4-GFP asymmetric distribution among mother and daughter cell cytoplasm was greatly disturbed in *atg18*Δ cells similar to the behaviour in *vac7*Δ cells, but at a lesser degree (figure 8*f*). Thus, PI3,5P_2_ modulates Kin4 cellular distribution mostly via Atg18 and probably also via additional mechanisms. Our data that deletion of *ATG18* does not completely prevent Kin4 vacuole localization in *fab1-ha* expressing cells (figure 8*a*) is also in support of this notion.

### 2.9. PI3,5P_2_ acts also through Atg18 and Kin4 independent means to promote timely mitotic exit

To understand whether PI3,5P_2_ function in mitotic exit is limited to Atg18-Kin4 pathway, we first analysed mitotic exit timing in the absence of Atg18. Cells lacking Atg18 are capable of producing PI3,5P_2_, even at higher levels than in wild-type cells [84]. Thus, differently from analysis of *vac7*Δ, analysis *of atg18*Δ cells allows for a condition where PI3,5P_2_ is present but PI3,5P_2_ dependent Kin4 regulation is impaired (figure 8*b* and *f*). Similar to *vac7*Δ, *atg18*Δ cells had prolonged anaphase (figure 8*g*) and similar to *VAC7* deletion, *ATG18* deletion in *lte1*Δ cells caused a drastic increase in anaphase duration (figure 8*h*). Likewise, deletion of *ATG18* was synthetic lethal with most of the *men-ts* mutants (electronic supplementary material, figure S8A; table 1). These data indicate that PI3,5P_2_-Atg18-Kin4 pathway is sufficient for effective stimulation of mitotic exit in the absence of PI3,5P_2_ overproduction (*fab1-ha)*. However intriguingly, over production of PI3,5P_2_ was able to accelerate mitotic exit in *atg18*Δ *lte1*Δ cells (figure 8*i*), indicating that PI3,5P_2_, when overproduced, is capable of accelerating mitotic exit independently of Atg18 in mitotic exit defective cells. Overproduction of PI3,5P_2_ was able to rescue growth of *mob1–67* in the absence of Atg18 which also supports this view (electronic supplementary material, figure S8B; table 1).

We next asked whether mitotic delay of *lte1*Δ*vac7*Δ cells can be rescued by *KIN4* deletion. Deletion of *KIN4* greatly decreased the anaphase duration of *lte1*Δ*vac7*Δ, however not to the levels of wild-type cells (figure 8*j*), suggesting that both Kin4-dependent and -independent mechanisms may account for the mitotic exit delay of *lte1*Δ *vac7*Δ cells. In line with this model, deletion of *KIN4* could only partially rescue the synthetic lethality of *cdc15–1 vac7*Δ cells (electronic supplementary material, figure S8C; table 1). These data altogether suggest that PI3,5P_2_ promote mitotic exit not only by Atg18-Kin4 pathway but also by Atg18 and/or Kin4 independent mechanisms. Of importance the latter becomes evident in *fab1-ha* expressing cells that over produce PI3,5P_2_ or in cells with mitotic exit defects such as *lte1*Δ and *men-ts* mutants.

## 3. Discussion

Phosphorylated phosphatidylinositol derivatives (phosphoinositides) are evolutionary conserved signalling lipids that recruit effector proteins to specific cellular membrane locations. Phosphatidylinositol-3,5-bisphosphate (PI3,5P_2_) is specifically synthesized on vacuole/lysosome membranes through phosphorylation of PI3P by Fab1 kinase [64,66]. In this study, we show that PI3,5P_2_ plays a role in timely execution of mitotic exit.

### 3.1. PI3,5P_2_ promotes mitotic exit via the PI3,5P_2_ binding protein Atg18 and mitotic exit inhibitor Kin4

Our findings indicate that PI3,5P_2_ has a mitotic exit promoting function which works mostly through the PI3,5P_2_ binding protein Atg18 and the mitotic exit inhibitor Kin4 (figure 8*k*). Several observations led to this conclusion. Firstly, disruption of the Fab1-Vac7-Vac14 complex caused lethality in cells with reduced mitotic exit activity. Secondly, a hyperactive allele of *FAB1* (*fab1-ha*) which leads to over production of PI3,5P_2_ rescued the prolonged anaphase and lethality of the mitotic exit mutants. Thus, Fab1's ability to produce PI3,5P_2_ correlates with cells' ability to exit mitosis. In addition, both overproduction of PI3,5P_2_ (*fab1-ha*) and lack of PI3,5P_2_ (*vac7*Δ) disturbed Kin4 localization. More specifically, elevated levels of PI3,5P_2_ caused Kin4 to mis-localize at the vacuole periphery, whereas in the absence of PI3,5P_2_ asymmetric distribution of Kin4 among mother and daughter cell cytoplasm was disrupted. In cells over producing PI3,5P_2_, Kin4 was unable to regulate Bfa1 localization, indicating impaired Kin4 function. Consistent with the impairment of Kin4, PI3,5P_2_ over producing cells showed deficiency in the SPOC, which heavily relies on Kin4 and Bfa1-Bub2 functionality. Notably, *fab1-ha* driven Kin4 vacuole recruitment and impairment of Kin4 function required the PI3,5P_2_ binding protein Atg18. Without Atg18, *fab1-ha* expression did not cause SPOC deficiency. Furthermore, similar to *vac7*Δ cells, *atg18*Δ cells had more symmetric distribution of Kin4 in the mother and daughter cell cytoplasm. These data suggested that PI3,5P_2_ impairs localization and function of the mitotic exit inhibitor Kin4 through the PI3,5P_2_ binding protein Atg18 (figure 8*k*).

### 3.2. How do PI3,5P_2_ and Atg18 impair Kin4 function?

Kin4 exhibits a very dynamic localization during the cell cycle [26–28,78]. It localizes to the mother cell cortex most of the time. For a short period during mid-to-late anaphase, Kin4 also localizes to the spindle pole body (SPB) that resides at the mother cell. At around cytokinesis, Kin4 localizes to the bud neck. Even though Kin4 appears to be mostly mother cell localized based on cortex and SPB localization, there is also a gradient of cytosolic Kin4 along the polarity axis, Kin4 being more concentrated at the mother than daughter cell cytoplasm [39]. Spatial arrangements of the MEN and SPOC components are critical for mitotic exit control [5,36]. Mitotic exit activating mechanisms including Lte1 are restricted to the daughter cell to create a mitotic exit activating zone therein, whereas in the mother cell a mitotic exit inhibitory zone is generated by Kin4 [36,39,41,85]. Accordingly, presence of Kin4 on the SPB in the daughter cell or increased overall levels of Kin4, which also increases its levels in the daughter cell, causes mitotic exit delay [26,27,39]. We propose that PI3,5P_2_ assists in retaining Kin4 within the mother cell or excluding it from the daughter cell, through an unknown mechanism that is highly depended on PI3,5P_2_ binding protein Atg18. Given that Kin4 co-localizes with vacuolar membranes and the mother cell cortex, roles of PI3,5P_2_ and Atg18 in membrane trafficking, endosome recycling and protein sorting [58,86] may account for the function of PI3,5P2-Atg18 pathway in establishment and/or maintenance of Kin4 asymmetrical distribution along the cell.

### 3.3. Does PI3,5P_2_ directly affect Kin4?

We do not yet know whether PI3,5P_2_ and/or Atg18 directly binds Kin4 to regulate Kin4 cellular distribution and function. We performed pull-down experiments from yeast lysates using PI3,5P_2_ coated beads, however Kin4 was not detected on these beads through mass-spectrometry analysis (data not shown). We also failed to observe Kin4-Atg18 interaction in co-immunoprecipitation experiments using cells expressing *fab1-ha* (data not shown). It is worth mentioning that Kin4 is a highly insoluble protein [41]. Most Kin4 was found in the insoluble fraction of cell lysates together with Vph1, the lysosomal V-ATPase [41]. This result supports colocalization of Kin4 with lysosomal membranes in cell lysates and supports our model that the lysosomal signalling lipid PI3,5P_2_ regulates Kin4. It also suggests that the soluble pool of Kin4 used in our pool down experiments may lack the form of Kin4 that is capable of binding to PI3,5P_2_ and/or Atg18. Nevertheless, we cannot rule out the possibility that Kin4 may require other proteins or lipids to bind to the lysosomal membrane. To understand how Kin4 binds to the vacuole membrane and whether it physically interacts with PI3,5P_2_ or Atg18, it is imperative to conduct further experiments.

Atg18, is a conserved protein that binds PI3,5P_2_ and to some extent to PI3P to mediate crucial roles in cellular processes such as autophagy, membrane fission, cytoplasm to vacuole targeting (CVT) and regulation of PI3,5P_2_ synthesis [50,79,84,87–89]. Atg18 creates a negative feedback inhibition on PI3,5P_2_ synthesis, thus it contributes to the transient nature of PI3,5P_2_ signaling. Roles of Atg18 in vacuole membrane fission and retrograde membrane traffic from vacuole to Golgi via endosomes, but not in autophagy or CVT appears to be dependent on PI3,5P_2_ [79,84]. Atg18 also plays a role in blockage of PI3,5P_2_ dependent nuclear division in response to methylglyoxal, a natural metabolite derived from glycolysis [90]. Our work suggests that Atg18 becomes crucial for PI3,5P_2_ dependent activation of mitotic exit through regulation of Kin4. Therefore, it is tempting to speculate that PI3,5P_2_ employs Atg18 to drive at least some of its functions.

### 3.4. Does PI3,5P_2_ promote mitotic exit only via Atg18 dependent Kin4 impairment?

Atg18 dependent Kin4 impairment appears not to be the only way by which Fab1 promote mitotic exit. First, modulation of Kin4 by PI3,5P_2_ probably requires other factors in addition to Atg18. Data that support this notion are (1) Kin4 asymmetric distribution in mother and daughter cell cytoplasm was disturbed in *atg18*Δ, similar to but slightly milder than *vac7*Δ. (2) Albeit greatly reduced, Kin4 vacuole periphery localization did not completely disappear in *fab1-ha* expressing *atg18*Δ cells. Second, Kin4 activity does not fully account for the mitotic exit delay of *vac7*Δ *lte1*Δ cells as deletion of *KIN4* in *vac7*Δ *lte1*Δ only partially rescued the prolonged anaphase duration. In line with this data, deletion of *KIN4* only partially rescued lethality of *cdc15–1 vac7*Δ cells. Thus, in addition to the Atg18-Kin4 pathway Fab1-PI3,5P_2_ may promote mitotic exit by other mechanisms (figure 8*k*). These additional mechanism are likely to be at the level of or upstream of the MEN, rather than a bypass of the MEN, as *fab1-ha* does not promote growth of MEN mutants at their restrictive temperatures.

### 3.5. Does PI3,5P_2_ promote mitotic exit in every cell cycle?

We used mutants defective in mitotic exit to analyse the contribution of PI3,5P_2_ to mitotic exit. Impairment of PI3,5P_2_ synthesis through deletion of *VAC7*, *VAC14* or usage of a temperature sensitive allele of Fab1 suppressed the growth of mitotic exit mutants, whereas elevation of PI3,5P_2_ levels through usage of a hyperactive Fab1 allele (*fab1-ha*), improved growth of mitotic exit mutants. Based on anaphase duration calculations in *lte1*Δ cells, mitotic exit was delayed upon deletion of *VAC7* whereas it was accelerated upon expression of *fab1-ha*. Also, *fab1-ha* decreased the proficiency of SPOC, which is a mitotic checkpoint that creates a STOP mitotic exit signal. Therefore, all these aforementioned experiments reflected conditions with *a priori* mitotic exit defects. In wild-type cells that do not have *a priori* mitotic exit defects, increased PI3,5P_2_ synthesis via *fab1-ha* expression did not accelerate mitotic exit. This raises the key question of whether PI3,5P_2_ signaling promote exit from mitosis only in cells with defective mitotic exit. Our data that *KIN4* deletion rescued the mitotic exit delay of *vac7*Δ cells strongly suggests that PI3,5P_2_ dependent inhibition of Kin4 is also required for timely mitotic exit in cells where the MEN is not compromised. In line with this notion, deletion of *VAC7* disturbed Kin4 cortex localization and asymmetric distribution of Kin4 in the mother and daughter cell cytoplasm. It is also worth mentioning that accelerating mitotic exit in wild-type cells is not an easy task due to the involvement of several levels of mechanisms in mitotic exit control. Indeed, deletion of well-characterized mitotic exit blockers such as *BFA1*, *BUB2* or *KIN4* cannot accelerate mitotic exit in an unperturbed cell cycle, either [78]. Likewise, despite presence of many essential proteins in the MEN, only overexpression of Cdc5 [91] or a dominant-active Cdc15 mutant [92] have been shown to achieve mitotic exit acceleration [93]. Therefore, other pathways most probably hinder overproduced PI3,5P_2_ from expediting mitotic exit in wild-type cells with a fully functional MEN, which may account for why *fab1-ha* expression does not accelerate mitotic exit in wild-type cells under normal conditions. Thus, we favour a model where PI3,5P_2_ dependent modulation of Kin4 cellular distribution works in every cell cycle for timely regulation of mitotic exit. PI3,5P_2_ regulation of Kin4 probably works in parallel to Lte1 to limit Kin4 activity in the daughter cell.

### 3.6. PI3,5P_2_, hyperosmotic stress and mitotic exit

PI3,5P_2_ is the least abundant of the seven PIPs. Under basal conditions, PI3,5P_2_ constitutes only ∼0.05–0.1% of the total phosphatidylinositol lipids [58]. Cellular levels of PI3,5P_2_, however, rapidly and drastically increases in response to stress conditions such as hyperosmotic shock and certain cytokine and hormones like EGF, IL-2 and insulin [64,94–100]. In budding yeast, PI3,5P_2_ levels increase approximately 20 fold within the first 10 min of hyperosmotic shock and drop back to normal levels in about 30 min [64,95]. Interesting coincidence is that hyperosmotic stress also promotes exit from mitosis of MEN mutants [93]. This hyperosmolarity dependent mitotic exit was shown to be dependent on the high osmolarity glycerol (HOG) mitogen-activated protein (MAP) kinase pathway. Deletion of HOG pathway components causes synthetic lethality in the MEN temperature sensitive mutants and *lte1*Δ cells [93], which is similar to our observation on the PI3,5P_2_ lacking mutants. HOG-dependent mitotic exit upon hyperosmotic shock was suggested as a mechanism that assures entry into G1 under unfavourable conditions [93]. We think that PI3,5P_2_ dependent inhibition of Kin4 may be working similarly. Indeed, we observed that without Vac7, hyperosmolarity via salt treatment is not able to rescue lethality of the MEN mutants (unpublished data). Thus, the two pathways that are activated by hyperosmolarity, HOG pathway and PI3,5P_2_, may be related in terms of mitotic exit regulation. It is intriguing to investigate the interaction of the two pathways in regulating mitotic exit under hyperosmotic conditions. Further studies may shed light on the molecular mechanisms and provide a comprehensive understanding of this biological processes.

### 3.7. PI3,5P_2_ and cell cycle control

In budding yeast, pieces of vacuole are segregated from the mother cell to the daughter cell along actin cables far before segregation of the nucleus to daughter cell [101]. Intriguingly mutants in which vacuole segregation is blocked can divide and the resultant daughter cell that lacks the vacuole spends more time at G1 before entering the cell cycle (G1/S transition) for de nova vacuole synthesis [102–104]. Studies from the Weisman laboratory suggest that absence of vacuole and PI3,5P_2_ delays cell cycle entry in budding yeast [63,83,105]. Thus, only in the presence of a mature vacuole, cells can commit for a new cell cycle. In other words, absence of vacuole/PI3,5P_2_ delays cell cycle entry in budding yeast. In fission yeast too, PI3,5P_2_ was implemented in mitotic commitment [62]. These findings indicate that PI3,5P_2_ dependent signalling may act as part of a checkpoint mechanism that provides cells time for de nova vacuole synthesis and/or maturation before G1/S transition.

Here, in this study, we show that PI3,5P_2_ dependent mechanisms promote mitotic exit (M/G1 transition). We think that the mitotic exit control by PI3,5P_2_ could be a way to communicate vacuole segregation with mitotic exit. It is tempting to speculate that lack of vacuole segregation and thus lack of PI3,5P_2_ in the daughter cell compartment may delay mitotic exit to give cells time to complete vacuole segregation before mitotic exit. However, given that cells can complete cell division in the absence of vacuole segregation, this delay probably does not completely guarantee that vacuole segregation takes place before mitotic exit. Careful analysis of mitotic exit and vacuole segregation timing would be necessary to understand whether such communication exists.

Inhibitors of PI3,5P_2_ synthesis have shown promise as drugs against several cancers and neurodegenerative diseases [61,72,74]. Although blockage of autophagy and disruption of lysosome homeostasis is considered as some of the reasons, how inhibition of PI3,5P_2_ selectively kills cancer cells remains largely elusive. This study and others that demonstrated dependency of cell cycle progression on PI3,5P_2_ at multiple stages suggest an active link between the cell cycle of the budding yeast and PI3,5P_2_ signalling. Thus, despite differences in yeast and human cell division cycles, we think that similar mechanisms may employ in higher eukaryotes too given the conserved nature of cell biology.

## 4. Materials and methods

### 4.1. Yeast methods, strains and plasmids

All yeast strains used are isogenic with S288C and are listed in electronic supplementary material, table S1. Basic yeast methods and growth media were as described [106]. Carbon source is glucose unless otherwise stated. When indicated 2% D-Raffinose (raffinose containing medium) or 2% D-Raffinose and 3% D(+)-Galactose (galactose containing medium) were used instead of glucose. Yeast strains were grown at 30°C and in rich medium unless otherwise stated. Plasmid containing strains were grown in synthetic complete (SC) media lacking the appropriate auxotrophic nutrients. Met3-*CDC20* strains were grown in SC media lacking methionine and cysteine. The temperature sensitive mutants and *kar9*Δ cells were maintained at 23°C. Most *lte1*Δ, *vac7*Δ and *kar9*Δ were maintained through complementation with *URA3*-based centromeric plasmids (pRS316/pRS416) and were grown on 5-Fluoroorotic acid (5FOA) plates before analysis. 5FOA negatively selects for the *URA3*-based plasmid and allows for observation of the phenotype coming from gene deletion.

Cassette PCR-based gene editing method was used for chromosomal gene deletion and C-terminal or N-terminal tagging [107,108]. Plasmids used in this study are listed in electronic supplementary material, table S2. *GFP-TUB1*, mCherry-*TUB1* and *fab1-ha* containing integration plasmids were integrated into the corresponding auxotrophic marker on the chromosome.

### 4.2. Cell synchronization

For G1 arrest, log-phase cell cultures grown in YPAD were treated with 10 µg ml^−1^ alpha-factor (Sigma #T6901) until greater than 95% of the cells had mating projections. Cells were released from alpha factor arrest in fresh YPAD after washing 3 times. For nocodazole synchronization, cell cultures grown to log phase in YPAD, were treated with 15 µg ml^−1^ nocodazole (Sigma #M1404). Synchronization was confirmed by microscopy after Ethanol fixation followed by staining with 1 µg ml^−1^ 4′,6-diamino-2-phenylindole (DAPI, Sigma).

### 4.3. Overexpression and depletion experiments

For Cdc20 depletion and Kin4 overexpression experiments, log-phase cell cultured at 30°C in raffinose containing SC medium lacking methionine and cysteine (SC-Met-Cys/Raff) were resuspended in raffinose containing SC-complete medium supplemented with 2 mM methionine and 2mM cysteine and incubated at 30°C for approximately 3 h to allow Cdc20 depletion. After achievement of metaphase arrest, cell culture was split into 2 flasks. 2% glucose was added into one of the flasks, and 2% galactose was added into the other to allow suppression or induction of Gal1 promoter respectively. After 3 h incubation at 30°C, cells were collected for live imaging and total protein extraction. For depletion of Lte1, log-phase cultures grown in YP/Raff-Gal were transferred to YPAD media at 23°C. Samples were taken for eight hours for microscopy and total protein extraction.

### 4.4. Spot assay for cell growth comparison on agar plates

Yeast cultures were grown in appropriate media and growth conditions until stationary phase. The OD_600_ of the cultures were adjusted to 1 and 10-fold serial dilutions were made using sterile PBS. 10 µl of serial dilutions were spotted on appropriate agar plates and grown at appropriate temperatures for 1–3 days.

### 4.5. Fluorescent microscopy

All microscopy experiments were performed using the Carl Zeiss Axio Observer 7 motorized inverted epifluorescence microscope with Colibri 7 LED light source, Axiocam 702 monochrome camera, 63× Plan Apochromat immersion oil objective lens, Zeiss filter sets 95 (GFP/Cherry), 20 (Rhodamin), 44 (FITC) and 49 (DAPI), and an XL incubation and climate chamber. 13 z-stacks of 0.3 µm thickness were taken for each stage position.

For time lapse experiments, cells were grown in filter sterilized SC-complete media to the log phase and attached on glass-bottom Petri dishes (WVR 10810–054 Matsunami) before the experiment [109]. Briefly, center of the dish was covered with 6% Concanavalin A (Canavalia ensiformis Jack Bean, type IV Sigma C2010-G), then the excess was washed out using sterile water. 200 µl of logarithmically growing culture of 0.5–0.8 OD_600_ was added to the glass center of the dish, followed by incubation at 30°C for 30 min. Cells were aspirated, and the dish was washed with prewarmed media to discard any non-attached cells. The dish was then filled with media, taken to the microscope stage, and let sit for 1 h on the stage before time-lapse started. For timelapse movies acquired at 30°C, microscope chamber was heated to 30°C 2–3 h in advance. If time-lapse was done at a cold temperature of 21°C, all incubations were done at room temperature and cells were washed with room temperature media. In that case, the microscope room was set to a temperature of 21°C and the microscope climate chamber was removed.

Samples collected from the time course experiments were fixed using 70% ethanol at each time point and kept at 4°C. For staining, cells were centrifugated at 3200 rpm for 2 min, then resuspended in the appropriate amount of PBS containing 1 µg ml^−1^ DAPI.

Staining of the vacuole with FM4–64 was performed as described [110] with slight modification. Briefly, 1 ml of log phase culture (0.5–0.8 OD_600_) grown in YPAD was centrifuged at 3200 rpm for 2 min. Cells were resuspended in 50 µl of YPAD containing 1–2 µl of the 40 µM FM4–64 solution and incubated at 30°C for 30–40 min, shaking. 1 ml YPAD was added and centrifuged at 32 000 rpm for 2 min. Cell pellets were washed twice with SC-complete filter sterilized medium. Finally, cells were resuspended in an appropriate amount of SC-complete filter sterilized medium and imaged.

Still images of Kin4-GFP bearing cells were taken without cell fixation using the 63x objective and 2 × 2 binning. Briefly, 1 ml of log-phase culture (0.5–0.8 OD_600_) grown in SC-complete was centrifuged at 3200 rpm for 2 min and cells were resuspended in appropriate volume of SC-complete medium.

### 4.6. Fluorescence intensity quantifications of Kin4-GFP in the mother and daughter cell compartments

Mean fluorescence intensities of Kin4-GFP at the mother and daughter cell cytoplasm were measured using Image J (NIH) mean fluorescence intensity measure tool from the sum projected microscopy images. Images were carefully acquired taking one image per slide, with a longer exposure at the GFP channel (800 msec) than normally performed. 13 z-stacks of 0.3 µm was taken for each image. Z-stacks were sum projected using the Image J z-projection tool.

Areas to be measured were carefully chosen at the GFP channel, from regions that has homogeneous signal distribution (away from very bright or very dark regions in the cell) within the mother and daughter cell compartments. As background reference, a cell free area outside of the cell was chosen. The mean signal intensities were corrected against the background signal by subtracting the background signal from the signal of the region of interest. For each cell, corrected mean signal intensity of the mother was divided to that of the daughter to obtain the mother-to-daughter signal ratios.

### 4.7. Protein methods

The total yeast cell protein precipitation, sample preparation and western blotting were performed as described [111]. Primary antibodies used in this study are mouse-anti-HA (gift from Gislene Pereira), rabbit-anti-tubulin (Abcam EPR13799), mouse-anti-myc (gift from Gislene Pereira), rabbit-anti-Clb2 (gift from Gislene Pereira) and rabbit-anti-Kin4 (gift from Gislene Pereira). Secondary antibodies were goat-anti-mouse (Advansta #R-05071-500) or goat-anti-rabbit (Advansta #R-05062-500) horseradish peroxidase (HRP) conjugated secondary antibodies. The chemiluminescence signals were detected using Biorad Chemidoc MP. Clb2 and Tubulin band intensities were measured using Image J (NIH) and corrected for the membrane background signal. Area sizes measured were kept constant between each time point. Relative Clb2 levels were calculated by dividing background corrected Clb2 band intensities to the corresponding background corrected Tubulin band intensities.

### 4.8. SPOC deficiency assay

*kar9*Δ cells containing GFP-Tub1 were cultured at 23°C till the log-phase and then incubated at 30°C for 5 hours. After 5 h, cells were imaged without fixation. Cells with correctly positioned anaphase spindles, mispositioned anaphase spindles and SPOC-deficient phenotypes (broken spindle in one cell body, more than one spindle in one cell) were counted. SPOC deficiency index was calculated as described [33] using the following formula:

SPOC deficiency index: (% cells with SPOC phenotype)/(%cells with misaligned spindle)×10.

### 4.9. Anaphase duration and spindle elongation dynamics

Spindle lengths were measured at each time point using Image J (NIH) measure tool, from the maximum projected timelapse series. Measured spindle lengths from different cells of the same population were aligned according to the anaphase onset (t6 in electronic supplementary material, figure S3) and a mean spindle length graph was plot for each strain. The time when spindle elongates were divided into three phases according to the slope of the curve. Slop of the three phases were calculated and compared. Anaphase duration was calculated as the time elapsed between the start of fast spindle elongation phase (PI) and spindle breakdown.

### 4.10. Statistical analysis

One-way ANOVA or unpaired two-tailed *t*-tests were applied to yield the significance differences between the samples using Prism 9.4.1 (GraphPad). For line fitting and slope comparison, simple linear regression analysis was performed in Prism 9.4.1.

## Data Availability

All data are presented in the main and supplementary figures [112]. Source data and raw images will be provided upon request as supplementary data.
